# Supplementary material for: Microglial﻿ signalling pathway deficits associated with the patient derived R47H TREM2 variants linked to AD indicate inability to activate inflammasome
Source: Sci Rep. 2021 Jun 25;11:13316. doi: 10.1038/s41598-021-91207-1 (PMC8233372; doi:10.1038/s41598-021-91207-1)

# Supplementary File

# Microglial signalling pathway deficits associated with the patient derived R47H TREM2 variants linked to AD indicate inability to activate inflammasome

Katharina Cosker, Anna Mallach, Janhavi Limaye, Thomas M Piers, James Staddon, Stephen J Neame, John Hardy, Jennifer M Pocock*

**Supplementary Files Figure Legends**

**Supplementary Figure 1. i** Flow cytometry of Annexin-V/propidium iodide labelled SH-SY5Y cells following 2 h heat-shock at 45°C (left) and mean fluorescent intensity (right); data are mean ± SEM, n=3-9 cell lines from 3 individual experiments, ****$P$<0.0001 (two-way ANOVA with Tukey's correction). **ii.** Immunostaining of Annexin-V, propidium iodide and merged images of heat-shocked SH-SY5Y cells. Scale bar, 20 μm. **iii.** Flow cytometry of phagocytosis of DiI-labelled heat-shocked PS+ SH-SY5Y cells following 2 h incubation with iPS-Mg in control and T66M$^{hom}$ lines, with cytochalasin D and unstained control; data are mean ± SEM, n=4-8 cell lines from 4 individual experiments, **$P$<0.01, ***$P$<0.001, ****$P$<0.0001 (one-way ANOVA with Tukey's correction), ns, non-significant. **iv.** Flow cytometry of phagocytosis of DiI-labelled heat-shocked PS+ SH-SY5Y cells following 2 h incubation showing no difference between isogenic lines control (BIONi-C) and R47H$^{hom}$ (BIONi-C7) lines, with cytochalasin D and unstained control; data are mean ± SEM, n=3-14 cell lines from 4 individual experiments, ****$P$<0.0001 (one-way ANOVA with Tukey's correction). **v.** ELISA of shed TREM2 in supernatant of iPS-Mg from control R47H$^{hom}$ lines with or without PS+ SH-SY5Y cells treatment and supernatant incubated with PS+ SH-SY5Y cells; data are mean ± SEM, n=4-8 cell lines from 4 individual experiments, *$P$<0.05 (one-way ANOVA). **vi.** Left, western blot analysis of TREM2 expression in iPS-Mg following 5 min stimulation with PS+ SH-SH5Y cells in control and TREM2 R47H$^{het}$ and R47H$^{hom}$ patient lines. Right, quantification of TREM2 protein normalised to beta-actin; data are mean ± SEM, n=2-15 cell lines from 3 individual experiments, not significant (two-way ANOVA with Tukey's correction).

**Supplementary Figure 2. i.** Left, western blot analysis of pAKT signalling in iPS-Mg following 5 min stimulation with PS+ SH-SY5Y cells or DOPS liposomes in control and TREM2 R47H$^{het}$ patient lines. Right, quantification of pAKT protein normalised to $^{beta}$-actin; data show mean ± SEM, n=3-4 cell lines from 3 individual experiments, *$P$<0.05, **$P$<0.01 (two-way ANOVA with Tukey's correction). **ii.** Left, western blot analysis of pAKT signalling in iPS-Mg following 5 min stimulation with PS+ SH-SY5Y cells or DOPS liposomes in control and TREM2 R47H$^{hom}$ patient lines. Right, quantification of pAKT protein normalised to beta-actin; data are mean ± SEM, n=2-4 from 3 individual experiments, not significant (two-way ANOVA with Tukey's correction).

**Supplementary Figure 3. i.** Caspase-1 activation in iPS-Mg treated with PS+ SH-SY5Y cells with or without the specific caspase-1 inhibitor YVAD-CHO; data are mean ± SEM, n=7 cell lines from 5 individual experiments, *$P$<0.05, ***$P$<0.001 (two-way ANOVA with Tukey's correction). **ii-iv.** Caspase-1 activation in iPS-Mg following treatment with **ii.,** NLRP3 (50 mM BAY11), **iii.,** SYK (500 nM PRT) or **iv.,** TLR4 (500 nM E5564) inhibitors and stimulation with overnight PS+ SH-SY5Y cells or overnight LPS + 30 min ATP in control, R47H$^{het}$ and R47H$^{hom}$ lines; data show mean ± SEM, n=5-15 cell lines from 4 individual experiments.

**Supplementary Figure 4:** Each Fig1-3 and Suppl 1,Supp 2 is the full blot of the corresponding figure to which it refers in the main text or in the supplementary files

# Supplementary figure 1:

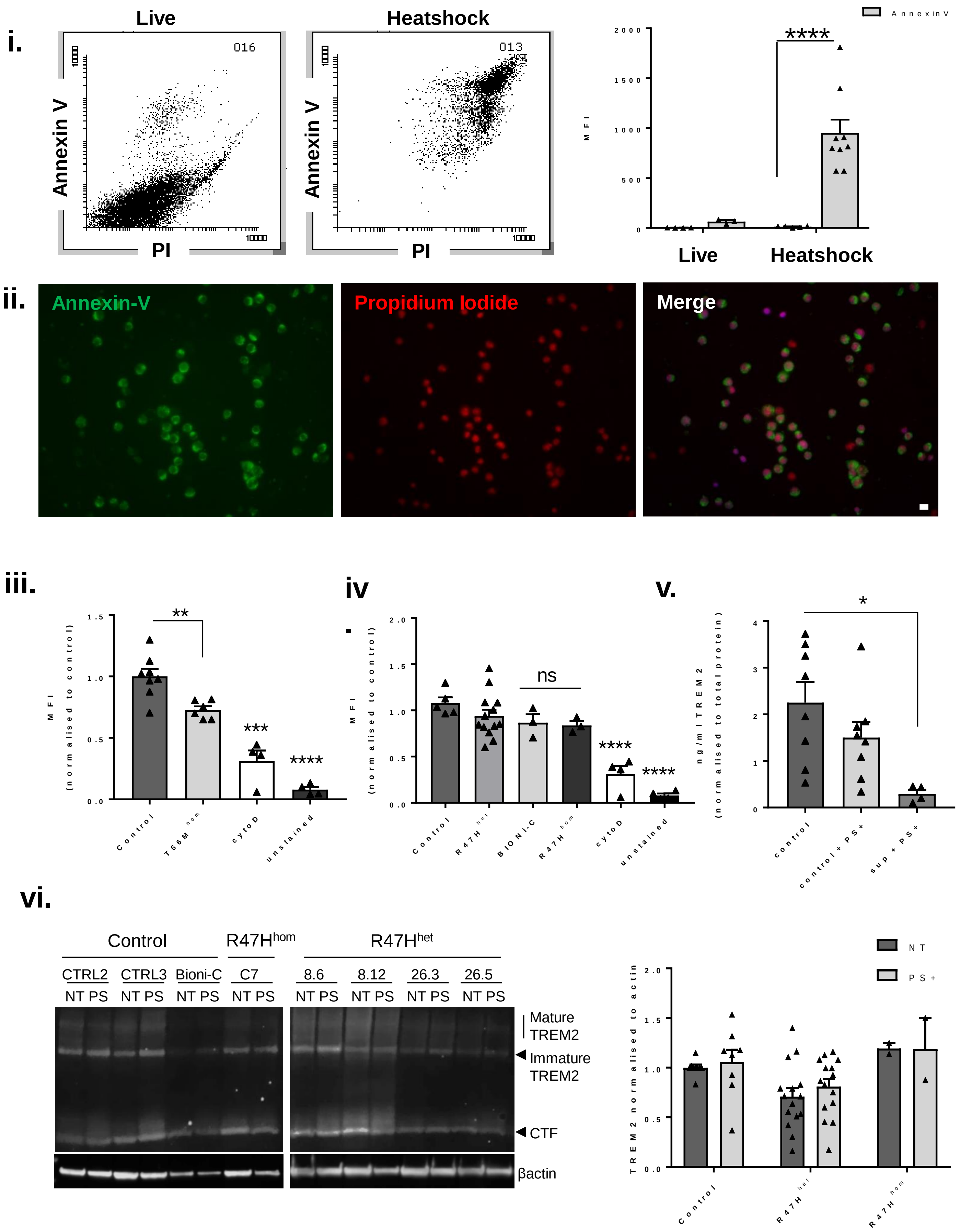

# Supplementary figure 2:

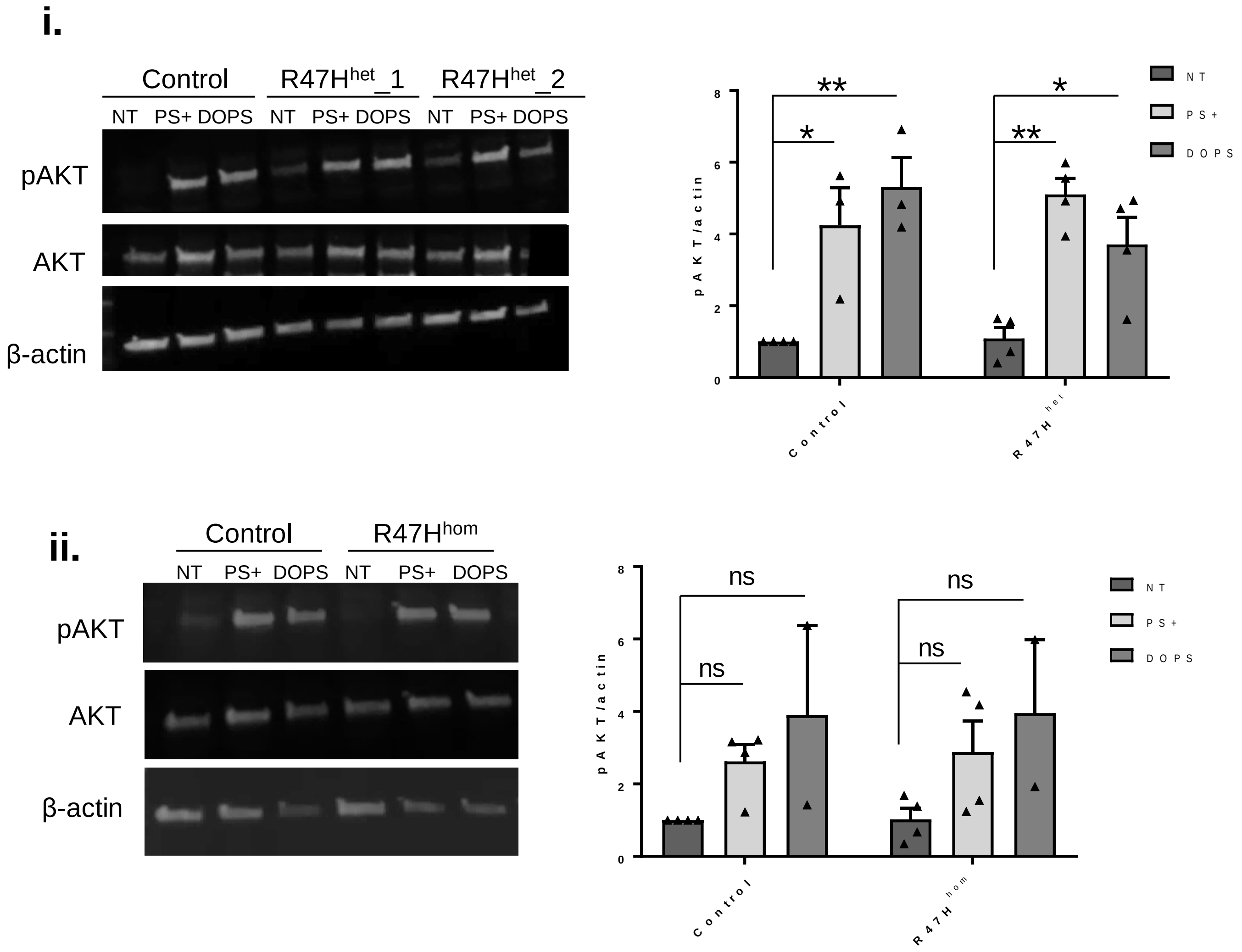

# Supplementary figure 3:

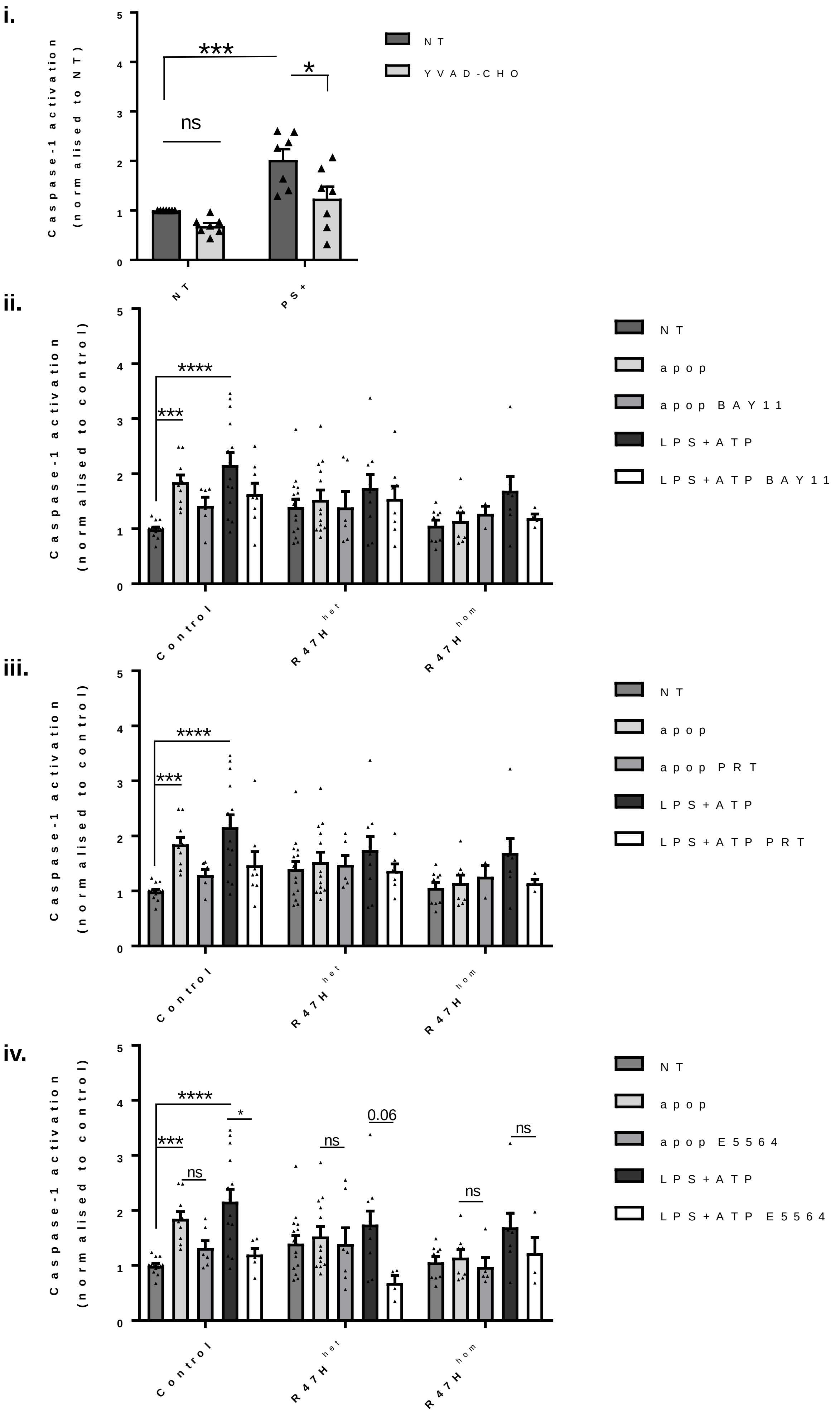

# Supplementary figure 4:

## Fig 1a.

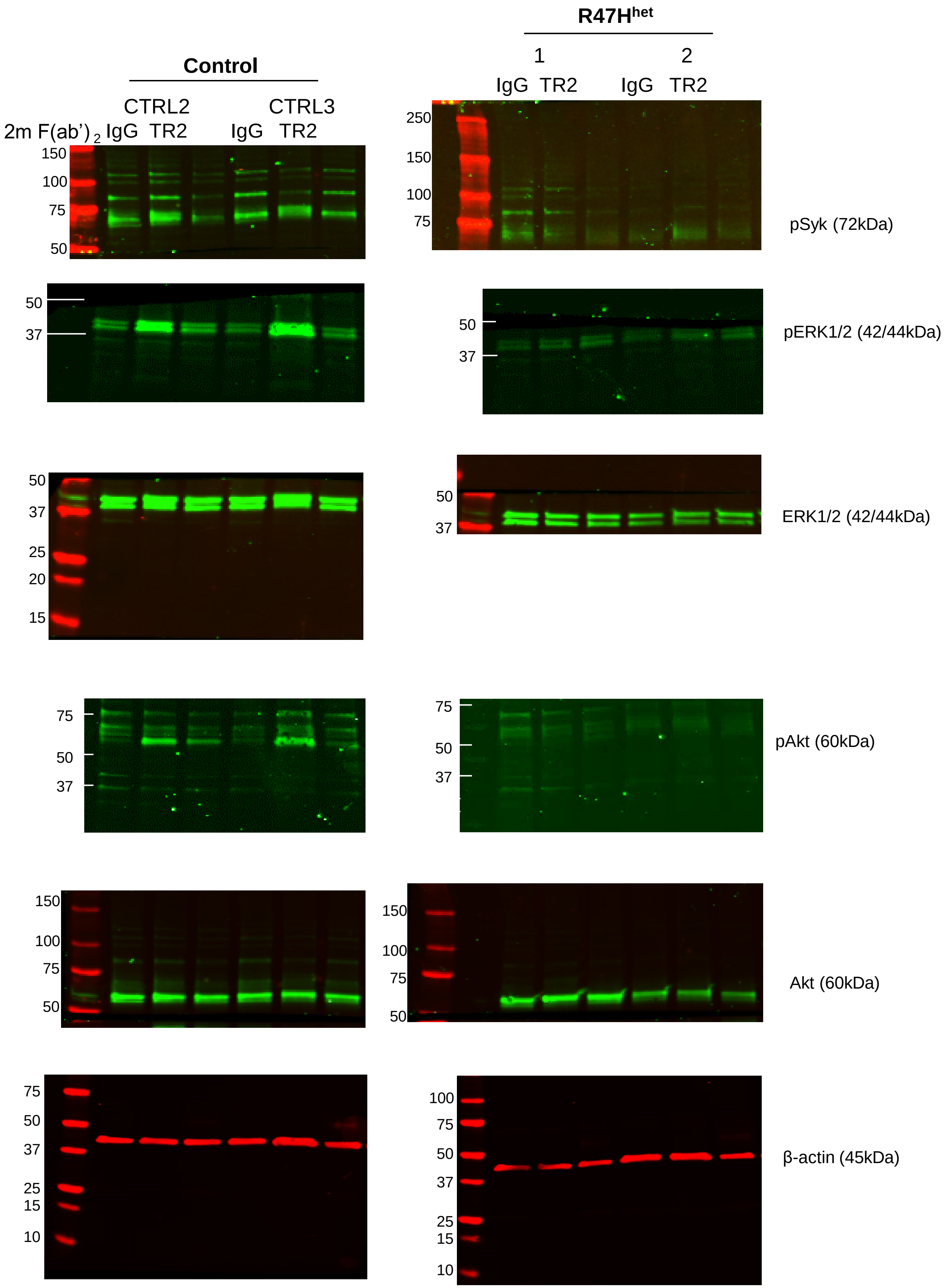

# Supplementary figure 4:

Fig 2.

c.

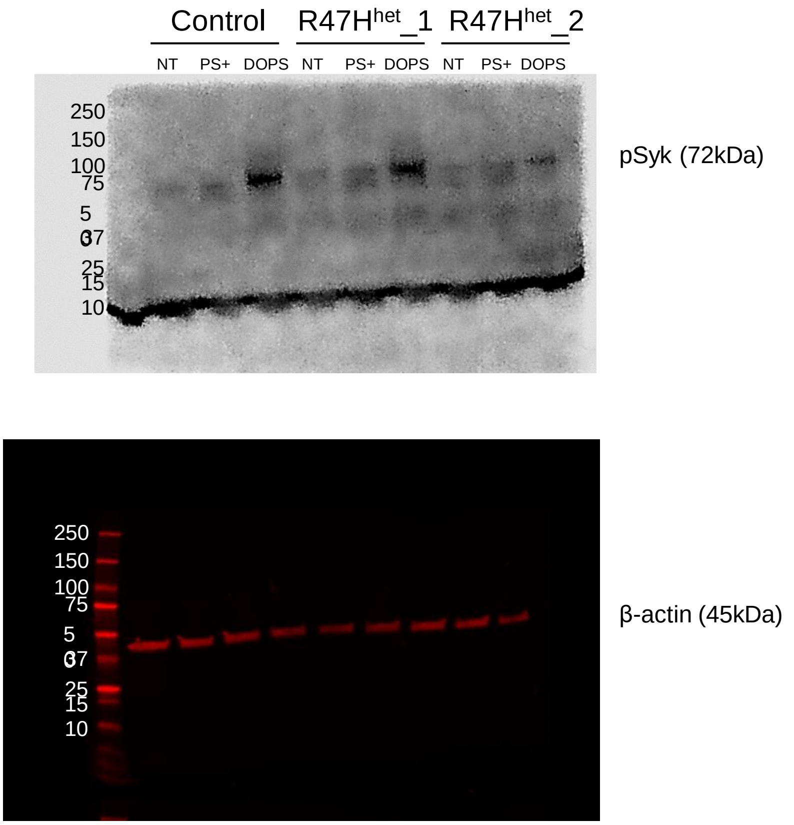


d.

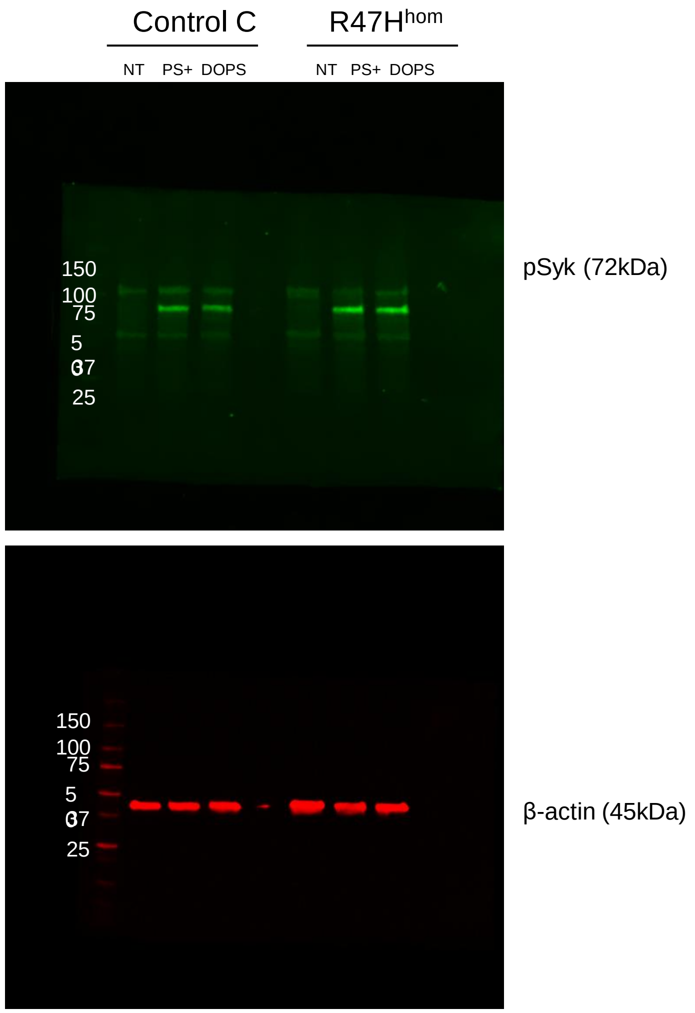

# Supplementary figure 4:

Fig 3.

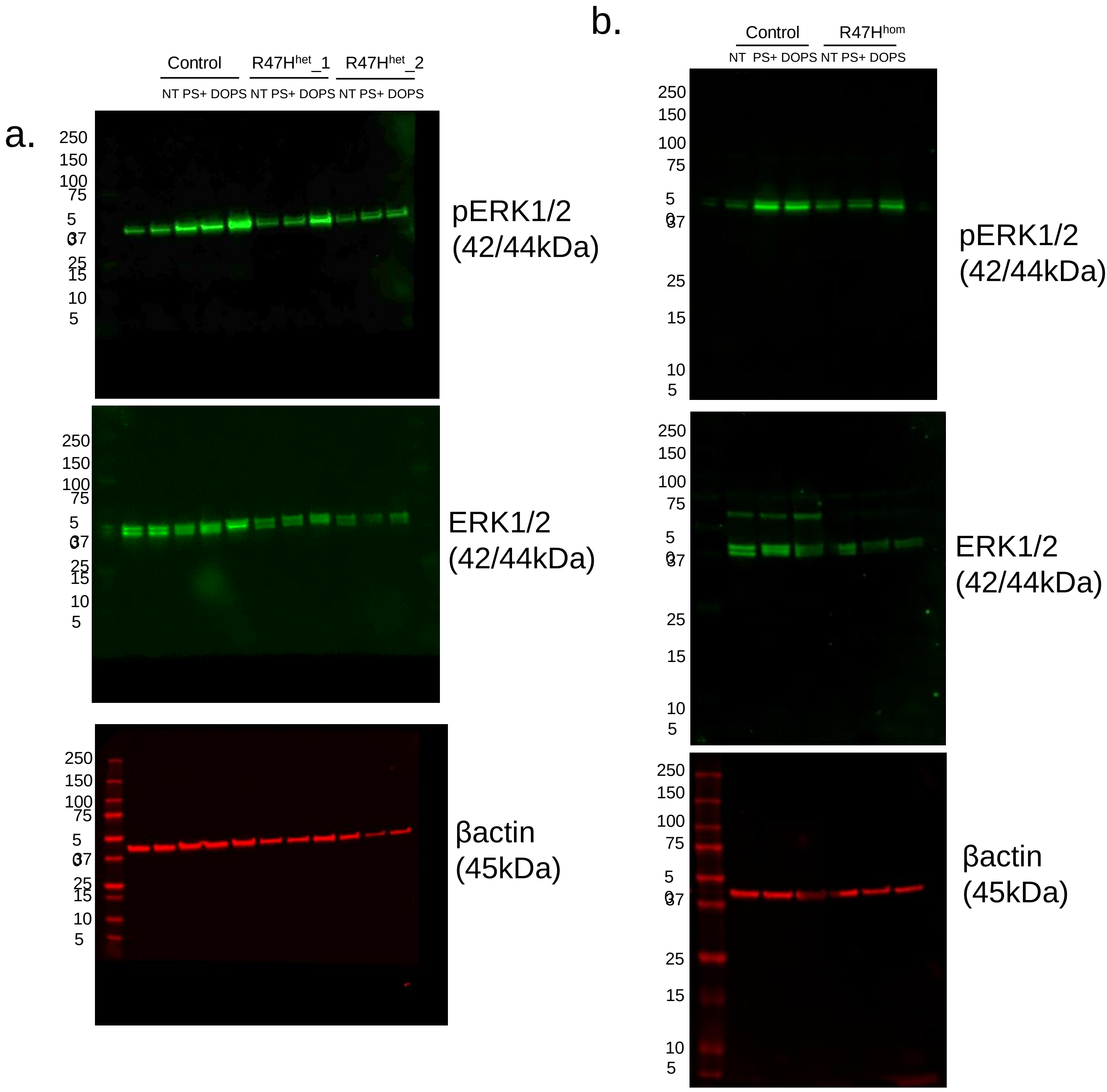

# Supplementary figure 4:

Fig 3.

c.

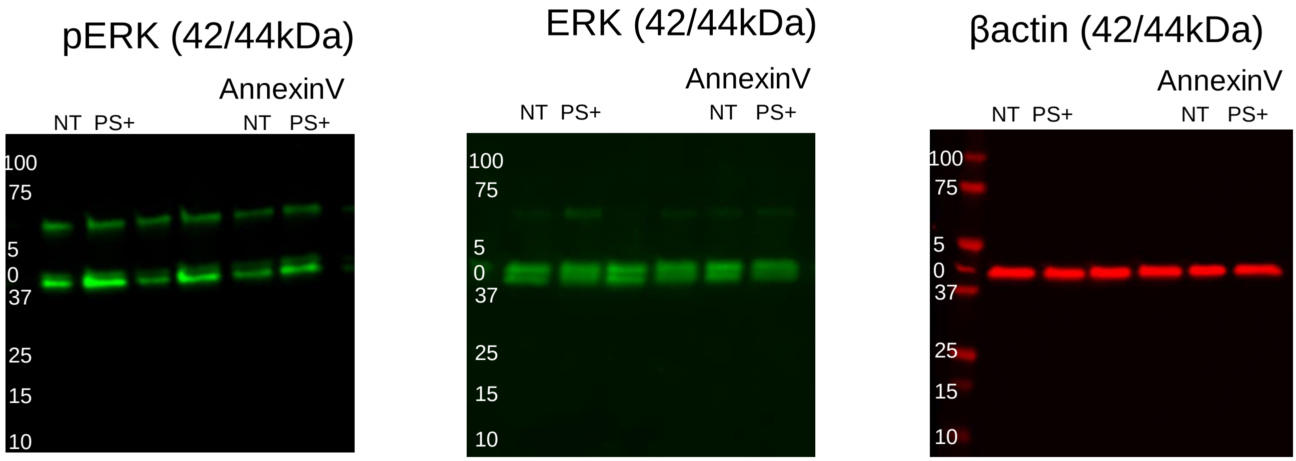


d.

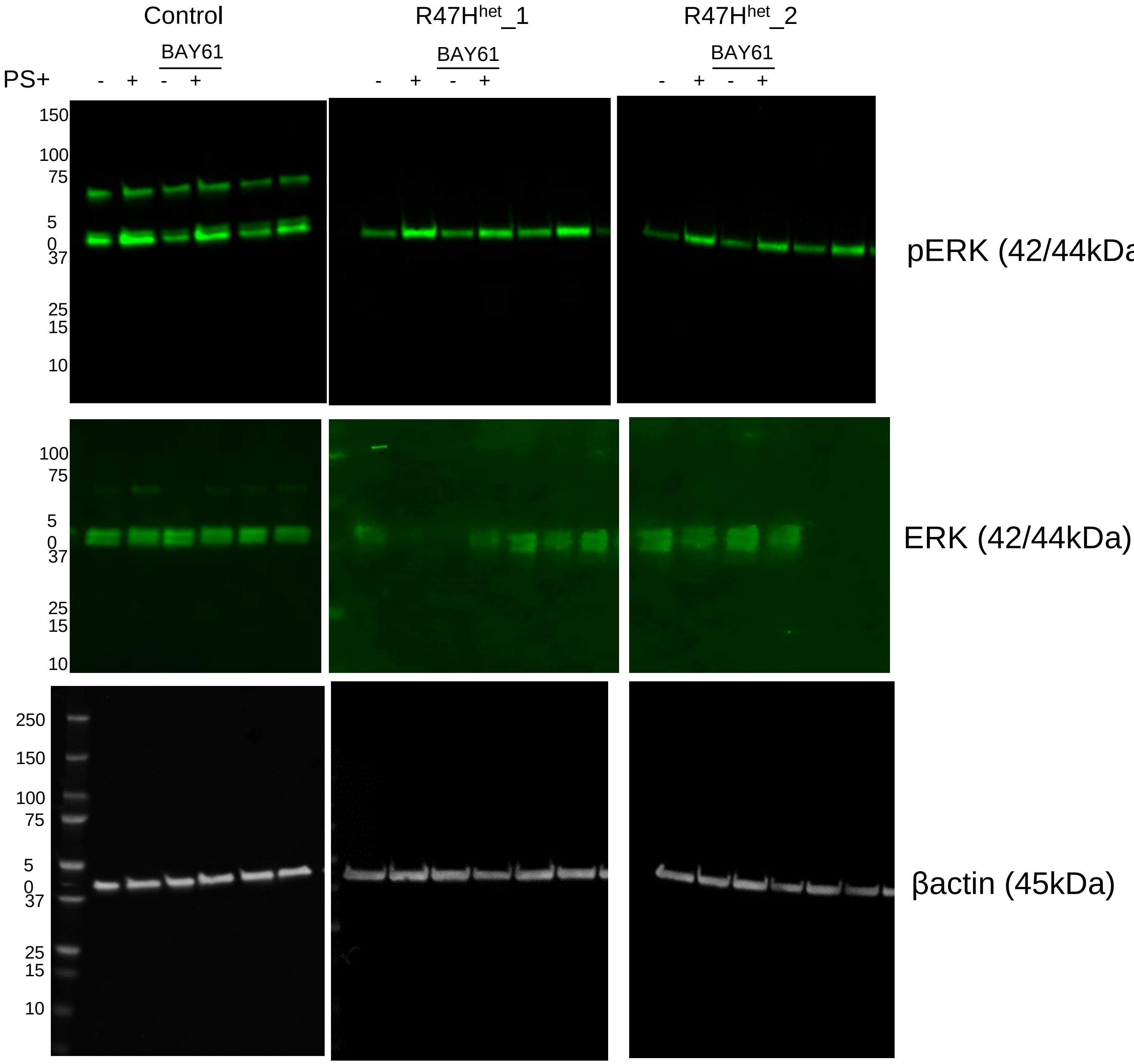

# Supplementary figure 4:

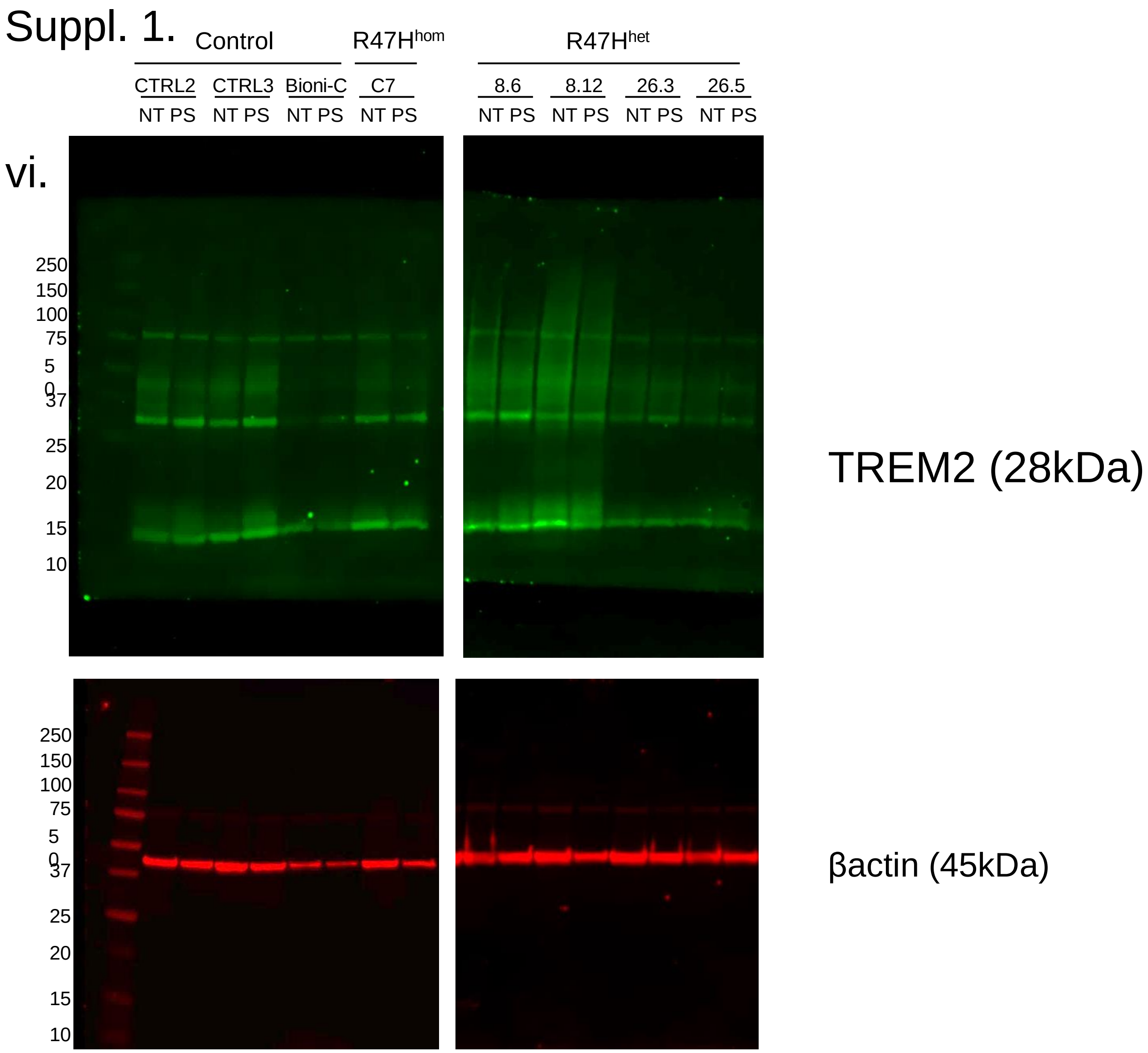

# Supplementary figure 4:

Suppl. 2.

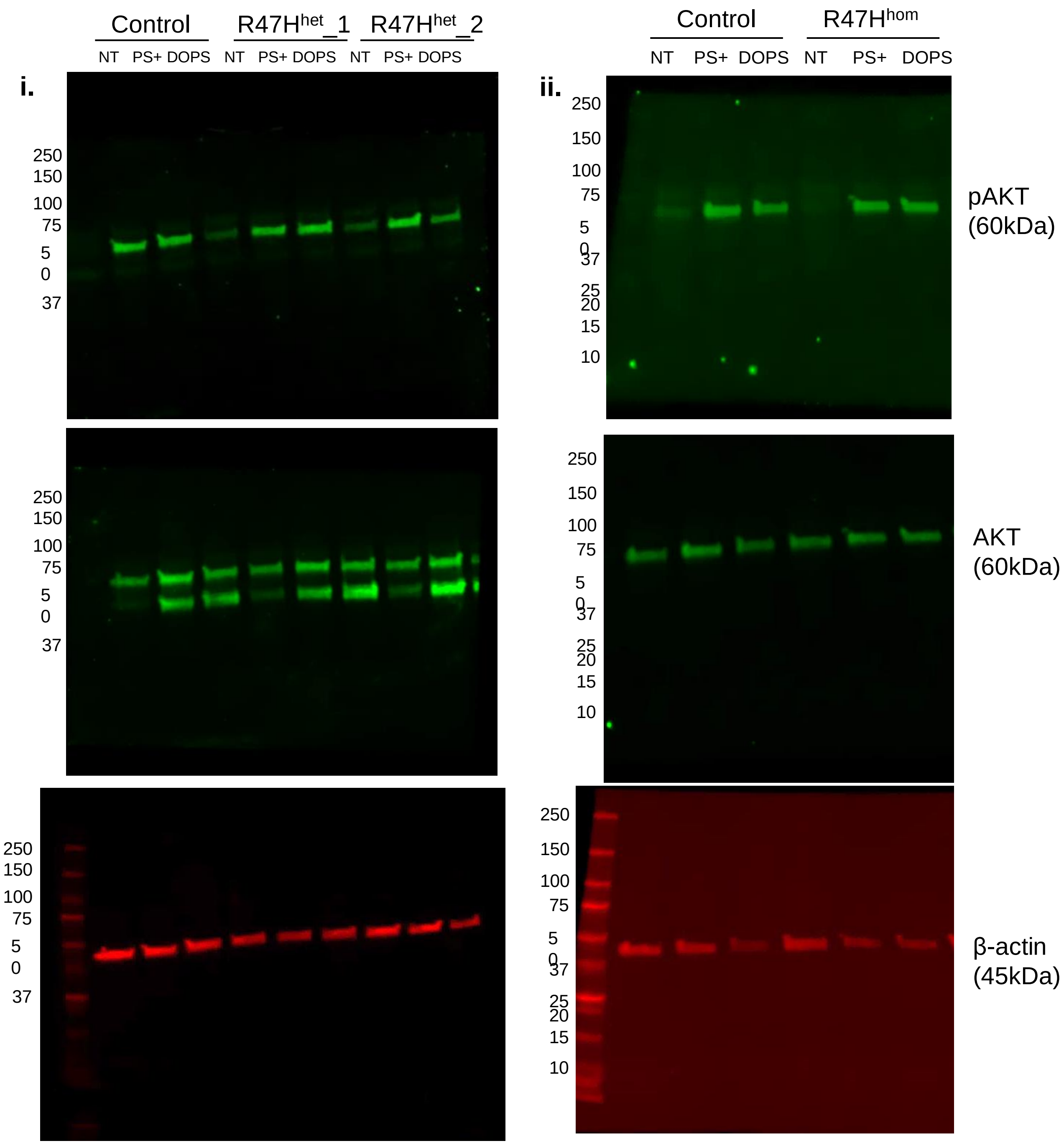

Supplement: Supplementary file 1 — Supplementary Information. [file 41598_2021_91207_MOESM1_ESM.pdf]
